# Association of dimethylarginines and mediators of inflammation after acute ischemic stroke

**DOI:** 10.1186/1742-2094-9-251

**Published:** 2012-11-17

**Authors:** Shufen Chen, Jens Martens-Lobenhoffer, Karin Weissenborn, Jan T Kielstein, Ralf Lichtinghagen, Milani Deb-Chatterji, Na Li, Anita B Tryc, Annemarie Goldbecker, Qiang Dong, Stefanie M Bode-Böger, Hans Worthmann

**Affiliations:** 1 Department of Neurology, Hannover Medical School, Carl-Neuberg-Strasse 1, Hannover, 30623 Germany; 2 Department of Neurology, Huashan Hospital, Fudan University, 12 Wulumuqi Zhong Road, Shanghai, 200040 China; 3 Department of Clinical Pharmacology, Otto-von-Guericke University of Magdeburg, University Hospital, Leipziger Strasse 44, Magdeburg, 39120 Germany; 4 Center for Systems Neuroscience (ZSN), Bünteweg 2, Hannover, 30559 Germany; 5 Department of Nephrology and Hypertension, Hannover Medical School, Carl-Neuberg-Strasse 1, Hannover, 30623 Germany; 6 Department of Clinical Chemistry, Hannover Medical School, Carl-Neuberg-Strasse 1, Hannover, 30623 Germany; 7 Department of Neurology, Beijing Tiantan Hospital, Capital Medical University, 6, Tiantan Xili, Beijing, 100050 China

**Keywords:** Asymmetric dimethylarginine (ADMA), Symmetric dimethylarginine (SDMA), Stroke, Inflammation

## Abstract

**Background:**

Elevated levels of asymmetric dimethylarginine (ADMA) and symmetric dimethylarginine (SDMA) are accompanied by endothelial dysfunction and predict adverse outcome after ischemic stroke. Via induction of oxidative stress, dimethylarginines are possibly linked to the inflammatory cascade after stroke that is known to considerably contribute to secondary progression of brain injury. We sought to investigate the association between dimethylarginines and inflammatory mediators in patients with acute ischemic stroke.

**Methods:**

Plasma levels of ADMA and SDMA were measured in prospectively collected blood samples of 58 patients with acute ischemic stroke. Blood samples were taken at 6 hours, 12 hours, 24 hours, 3 days and 7 days after onset of symptoms. Analyses of ADMA and SDMA were done by high-performance liquid chromatography-tandem mass spectrometry. Monocyte chemotactic protein-1 (MCP-1), matrix metalloproteinase-9 (MMP-9), tissue inhibitor of matrix metalloproteinase-1 (TIMP-1), interleukin-6 (IL-6), C-reactive protein (CRP) and S100B as markers of inflammation and brain damage were determined by commercially available immunometric assays. Patient data were compared with control data from 32 age-adjusted healthy volunteers. Baseline stroke severity was evaluated by the National Institutes of Health Stroke Scale (NIHSS) (NIHSS 0 to 1: mild stroke; NIHSS 2 to 8: moderate stroke; NIHSS ≥9: severe stroke).

**Results:**

Plasma ADMA and SDMA levels significantly correlated with blood levels of inflammatory mediators up to day 7 after stroke. On multiple stepwise linear regression analysis ADMA correlated with TIMP-1 at 6 hours, 24 hours, 3 days and 7 days, MMP-9 at 12 hours and IL-6 at 7 days (*P* <0.05) while SDMA correlated with MCP-1 at 6 hours, 24 hours, 3 days and 7 days as well as IL-6 at 3 days and 7 days (*P* <0.05).

**Conclusions:**

The levels of the vasoactive compound ADMA as well as levels of its structural isomer SDMA are associated with levels of inflammatory mediators after acute ischemic stroke. Further studies need to elucidate the cause and effect relationship of these crucial players.

## Background

The endogenous nitric oxide synthase (NOS) inhibitor asymmetric dimethylarginine (ADMA) is regarded as a mediator of oxidative stress and endothelial dysfunction. Increased levels of ADMA have been observed in patients with hypertension, hyperlipidemia, diabetes, old age and atherosclerotic burden [1, 2]. The concentration of ADMA increases after acute stroke in both plasma [3] and cerebrospinal fluid [4]. Symmetric dimethylarginine (SDMA), the structural isomer of ADMA, has been identified as a predictor of mortality after acute ischemic stroke [5]. Our group recently described that ADMA and SDMA plasma levels are independently associated with stroke outcome [6].

After occlusion of brain arteries and rapid depletion of substrates a large number of cellular and noncellular inflammatory events are initiated. Inflammation represents one of the predominant mechanisms for secondary injury after ischemic stroke [7–9].

ADMA acts as a mediator of oxidative stress via the inhibition and uncoupling of NOS [10] and thereby increases the expression of inflammatory genes. On the other hand, inflammation activates the protein arginine methyltransferases (PRMTs) and inhibits the dimethylarginine dimethylaminohydrolases (DDAHs) [11] resulting in increased levels of dimethylarginines [12].

Although the relationship between dimethylarginines and inflammation has been described in other diseases [13–15], so far no data exist for acute ischemic stroke. In the present study, we aimed to analyze the association of ADMA and SDMA with interleukin-6 (IL-6), monocyte chemotactic protein-1 (MCP-1), C-reactive protein (CRP), matrix metalloproteinase-9 (MMP-9), tissue inhibitor of matrix metalloproteinase-1 (TIMP-1) and S100B as markers and mediators of inflammation and brain cell damage in patients with acute ischemic stroke.

## Subjects and methods

### Study population

Between January 2007 and February 2009, 58 acute ischemic stroke patients who were admitted to the Department of Neurology at Hannover Medical School, Germany, within 6 hours after symptom onset were enrolled. The current patient cohort derived from a cohort of a former study [6]. Ischemic stroke was defined as an acute onset focal neurological deficit combined with neuroimaging evidence of cerebral infarction by cranial computed tomography (CCT) or magnetic resonance imaging (MRI). Exclusion criteria were history of malignant tumour, haemorrhagic stroke, transient ischemic attack (TIA) and systemic inflammatory disease with a serum CRP level >50 mg/l or need for intravenous antibiotic treatment.

Clinical and demographic data of the patients including age, gender, stroke etiology, body mass index (BMI), creatinine, estimated glomerular filtration rate (eGFR) (evaluated by CKD-EPI equation), glycated haemoglobin (HbA1c), thyroid stimulation hormone (TSH), treatment and vascular risk factors (hypertension, diabetes mellitus, hyperlipidemia, and smoking status) were recorded. Baseline stroke severity was evaluated by the National Institute of Health Stroke Scale (NIHSS) at admission according to the classification criteria of the NINDS rtPA Stroke Trial in 1995 [16]. Patients with NIHSS 0 to 1 at admission were considered as mild strokes, with NIHSS 2 to 8 as moderate strokes, while those with NIHSS ≥9 were classified into the severe group.

For comparison with the patient data, 32 healthy controls without malignant tumour and without any cardio-/cerebrovascular event at least during the previous two years were enrolled between April and July 2010. The study was approved by the local ethics committee. Patients and volunteers gave written informed consent.

### Blood sampling and marker quantification

Serum and EDTA-plasma samples were drawn from patients at 6, 12 and 24 hours, 3 and 7 days after onset of symptoms. Blood samples from controls were collected at enrolment. The samples were immediately centrifuged at 3000 r/min for 15 minutes. The supernatant was stored at −80°C until analysis.

ADMA and SDMA concentrations were assessed in EDTA plasma by high-performance liquid chromatography-tandem mass spectrometry as previously reported elsewhere [17]. IL-6, S100B and high-sensitivity CRP were determined in serum using the Immunolite 2000 (Siemens Healthcare Diagnostics, Eschborn, Germany) for IL-6, the Elecsys (Roche Diagnostics, Basel, Switzerland) for S100B and the BN II (Siemens Healthcare Diagnostics) for high-sensitivity CRP. MCP-1, MMP-9 and TIMP-1 were detected in EDTA plasma by enzyme-linked immunosorbent assay kits (R&D systems, Minneapolis, MN, USA). The inter-assay precision was 7.7% for ADMA, 4.9% for SDMA, <6.5% for IL-6, 5.0% for S100B, 4.0% for CRP, 8.0% for MMP-9, 7.0% for MCP-1 and 4.9% for TIMP-1. The intra-assay precision was 5.5% for ADMA, 3.9% for SDMA, <4.5% for IL-6, 2.1% for S100B, 4.6% for CRP, 10.0% for MMP-9, 7.8% for MCP-1 and 5.0% for TIMP-1.

### Statistics

Data were analysed using SPSS software package version 11.5 (SPSS Inc., Chicago, IL, USA). Baseline characteristics are shown as percentages or median with interquartile range. The differences of marker levels between stroke patients and controls were detected by one-way ANOVA. Within-group comparisons of marker levels at different time points were analysed by Friedman test and Wilcoxon test. The relation between levels of ADMA or SDMA and inflammatory markers was assessed by Pearson correlation analysis when the data were normally distributed and by Spearman rank correlation analysis when the data were not normally distributed. For significant correlations a multiple stepwise linear regression analysis adjusted for age, gender and stroke severity at admission was performed using the enter method. A probability value <0.05 was considered statistically significant.

## Results

### Demographics

The study population consisted of 58 patients with a median age of 72 years (interquartile range 58 to 77 years) and 32 healthy controls with a median age of 71 years (interquartile range 61 to 76 years). Demographic and clinical characteristics are shown in Table 1. Among the group of stroke patients, 22.4% had a mild stroke (NIHSS at admission 0 to 1), 55.2% had a moderate stroke (NIHSS 2 to 8), while 22.4% had a severe stroke (NIHSS ≥9). Patients suffered from atherothrombotic stroke in 20.7% of cases, cardioembolic stroke in 29.3%, lacunar stroke in 22.4% and stroke of undetermined cause in 27.6%.

**Table 1:** **Demographic and clinical characteristics of stroke patients and controls**

	Patients (n = 58)	Controls (n = 32)	***P***value
Age, years (mean and range)	72 (58, 77)	71 (61, 76)	0.999
Gender (female %)	44.8	56.3	0.299
Hypertension, %	56.9	75.0	0.088
Hyperlipidemia, %	41.4	53.1	0.284
Diabetes, %	10.3	12.5	0.739
Smoking, %	17.2	18.8	0.858
BMI, kg/m^2^	25.7 (24.5, 28.6)	25.6 (23.9, 30.0)	0.606
Creatinine, umol/L	78 (64, 86)	79.5 (72.3, 90.8)	0.367
eGFR, ml/min per 1.73 m^2^	80.6 (66.3, 93.1)	71.6 (62.3, 89.7)	0.123

Baseline characteristics are shown as percentages or median (interquartile range). BMI, body mass index; eGFR, estimated glomerular filtration rate.

### Time courses of ADMA, SDMA and inflammation markers

**Figure 1:**
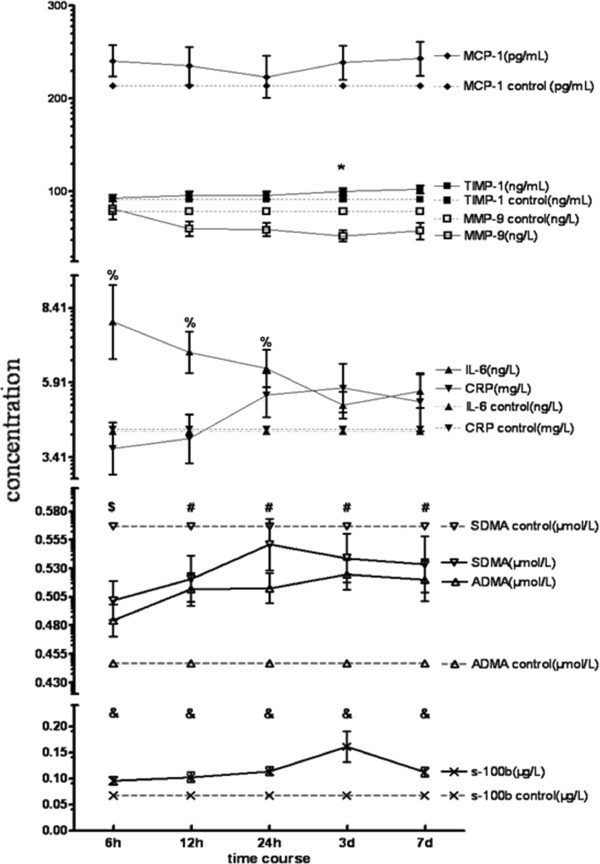
**Time courses of ADMA, SDMA, CRP, IL-6, S100B, MCP-1, TIMP-1 and MMP-9 after ischemic stroke.** Data are presented as mean and standard error. Between group comparisons of marker levels in patients and controls: *P <0.05: significant differences in TIMP-1 levels at 3 d. %*P* <0.05: significant differences in IL-6 levels at different time points. $*P* <0.05: significant differences in SDMA levels at 6 h. #*P* <0.05: significant differences in ADMA levels at different time points. &*P* <0.05: significant differences in S100B levels at different time points. Within-group comparisons of marker levels between baseline (6 h) and follow-up time points: significant differences (*P* <0.05) were detected for TIMP-1 (6 h vs. 3 d, 6 h vs. 7 d), MMP-9 (6 h vs. 12 h, 6 h vs. 24 h, 6 h vs. 3 d, 6 h vs. 7 d), IL-6 (6 h vs. 3 d, 6 h vs. 7 d), CRP (6 h vs. 12 h, 6 h vs. 24 h), SDMA (6 h vs. 12 h, 6 h vs. 24 h, 6 h vs. 3 d), ADMA (6 h vs. 12 h, 6 h vs. 24 h, 6 h vs. 3 d, 6 h vs. 7 d) and S100B (6 h vs. 24 h, 6 h vs. 3 d). ADMA, asymmetric dimethylarginine; CRP, C-reactive protein; IL-6, interleukin-6; MCP-1, monocyte chemotactic protein-1; MMP-9, matrix metalloproteinase-9; SDMA, symmetric dimethylarginine; TIMP-1, tissue inhibitor of matrix metalloproteinase-1.

The dimethylarginine (ADMA, SDMA) and inflammatory marker (CRP, IL-6, S100B, MCP-1, TIMP-1 and MMP-9) time courses after stroke onset are shown in Figure 1. ADMA levels at the time points 12 and 24 hours, 3 and 7 days, IL-6 levels at 6, 12 and 24 hours, and S100B levels at all time points were higher in stroke patients than in controls (*P* <0.05). SDMA levels at 6 hours and MMP-9 levels at 3 days were lower in stroke patients than in controls (*P* <0.05). Levels of MCP-1, CRP and TIMP-1 did not significantly differ between stroke patients and controls.

### Correlation of ADMA and SDMA with inflammatory markers

**Figure 2:**
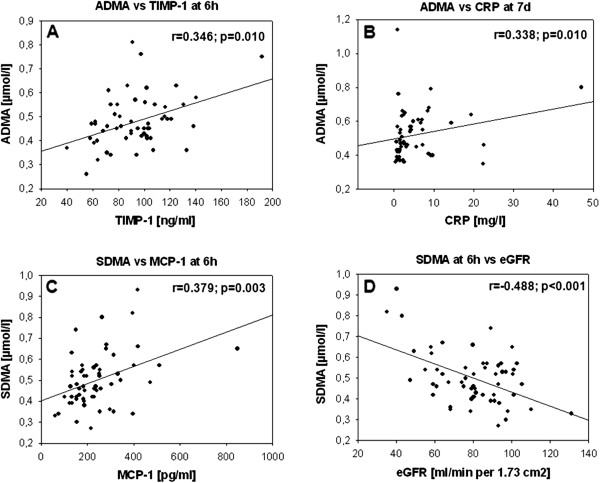
**Correlation of representative inflammatory markers and dimethylarginines after stroke onset.** (**A**) Correlation of ADMA and TIMP-1 levels at 6 h after stroke onset; (**B**) correlation of ADMA and CRP levels at 7 d after stroke onset; (**C**) correlation of SDMA and MCP-1 levels at 6 h after stroke onset; (**D**) correlation of SDMA at 6 h after stroke onset and eGFR. ADMA, asymmetric dimethylarginine; CRP, C-reactive protein; eGFR, estimated glomerular filtration rate; MCP-1, monocyte chemotactic protein-1; SDMA, symmetric dimethylarginine.

In stroke patients, levels of dimethylarginines were significantly correlated with levels of inflammatory markers at different time points (Table 2; Figure 2, A-C). ADMA was positively correlated with IL-6 and CRP at 3 and 7 days and with TIMP-1 at 6 and 24 hours, 3 and 7 days after stroke onset. SDMA was positively correlated with MCP-1 at 6 hours and 3 and 7 days, with IL-6 at 3 and 7 days and with TIMP-1 at 7 days after stroke onset. In controls dimethylarginines were not significantly correlated with inflammatory markers.

**Table 2:** **Correlation between dimethylarginines and inflammatory markers**

	6 h		12 h		24 h		3 d		7 d	
	**R**	**P**	**R**	**P**	**R**	**P**	**R**	**P**	**R**	**P**
ADMA vs. IL-6	0.059	0.659	−0.024	0.860	0.001	0.997	**0**.**272**	**0**.**039**	**0**.**344**	**0**.**008**
ADMA vs. CRP	−0.192	0.149	0.113	0.397	0.031	0.819	**0**.**291**	**0**.**027**	**0**.**338**	**0**.**010**
ADMA vs. S100B	−0.106	0.430	−0.073	0.585	0.144	0.279	0.082	0.540	0.214	0.106
ADMA vs. MMP-9	−0.104	0.440	−0.153	0.254	0.131	0.330	0.031	0.818	0.039	0.775
ADMA vs. TIMP-1	**0**.**346**	**0**.**010**	0.083	0.545	**0**.**401**	**0**.**002**	**0**.**431**	**0**.**001**	**0**.**352**	**0**.**008**
ADMA vs. MCP-1	0.179	0.179	−0.026	0.844	0.012	0.930	0.213	0.108	0.209	0.116
SDMA vs. IL-6	0.237	0.073	0.219	0.099	0.139	0.297	**0**.**389**	**0**.**003**	**0**.**405**	**0**.**002**
SDMA vs. CRP	−0.144	0.281	0.017	0.899	−0.023	0.863	0.207	0.119	0.197	0.138
SDMA vs. S100B	−0.110	0.410	0.033	0.808	0.135	0.313	0.036	0.788	0.182	0.170
SDMA vs. MMP-9	−0.062	0.649	−0.115	0.393	−0.132	0.329	−0.015	0.913	0.055	0.684
SDMA vs. TIMP-1	0.237	0.082	−0.015	0.914	0.048	0.726	0.170	0.215	**0**.**271**	**0**.**046**
SDMA vs. MCP-1	**0**.**379**	**0**.**003**	0.149	0.264	0.110	0.411	**0**.**286**	**0**.**030**	**0**.**345**	**0**.**008**

*P* <0.05 is considered statistically significant. ADMA, asymmetric dimethylarginine; CRP, C-reactive protein; IL-6, interleukin-6; MCP-1, monocyte chemotactic protein-1; MMP-9, matrix metalloproteinase-9; SDMA, symmetric dimethylarginine; TIMP-1, tissue inhibitor of matrix metalloproteinase-1.

SDMA but not ADMA was correlated with eGFR at each time point (*P* ≤0.001, Figure 2,D). Dimethylarginines did not correlate with HbA1c, TSH or age (*P* >0.05). The effects of emergency treatment using intravenous rtPA and concomitant medication including statins, angiotensin receptor blockers (ARB), angiotensin-converting enzyme (ACE) inhibitors and acetylsalicyclic acid (ASA) on ADMA and SDMA levels were investigated. Concomitant medication was defined as patients who were treated with these drugs prior to stroke event and during the study period, compared to those patients who were not treated either prior to the qualifying event or during the study period. No significant difference in ADMA and SDMA levels in regard to treatment was detected (*P* >0.05). Dimethylarginines on admission showed no significant differences in regard to stroke etiology (ADMA: *P* = 0.327; SDMA: *P* = 0.092).

As the inflammatory markers may affect each other, we tested their independent association with dimethylarginines by multiple stepwise linear regression analysis that was adjusted for age, gender, stroke severity at admission and the levels of inflammatory markers measured at the respective time point (and eGFR at baseline and stroke etiology for SDMA) (Tables 3 and 4). ADMA is positively correlated with TIMP-1 at 6 and 24 hours, 3 and 7 days after acute ischemic stroke onset. ADMA is inversely correlated with MMP-9 at 12 hours and positively correlated with IL-6 at 7 days. SDMA is positively correlated with MCP-1 at 6 and 24 hours, 3 and 7 days, with IL-6 at 3 and 7 days and with eGFR at each time point after stroke onset.

**Table 3:** **Independent determinants of plasma ADMA concentrations in acute ischemic stroke patients**

Time point	Parameters	β	SE	***P*** value
6 h	TIMP-1	0.412	0.001	0.002
12 h	MMP-9	−0.350	0.000	0.029
24 h	TIMP-1	0.426	0.000	0.002
3 days	TIMP-1	0.452	0.000	0.001
7 days	IL-6	0.629	0.003	0.000
	NIHSS 2-8	0.411	0.035	0.002
	NIHSS >8	0.159	0.041	0.216

Multiple stepwise linear regression analysis adjusted for age, gender, and stroke severity at admission. ADMA, asymmetric dimethylarginine; IL-6, interleukin-6; MMP-9, matrix metalloproteinase-9; NIHSS, National Institutes of Health Stroke Scale; TIMP-1, tissue inhibitor of matrix metalloproteinase-1.

**Table 4:** **Independent determinants of plasma SDMA concentrations in acute ischemic stroke patients**

Time point	Parameters	β	SE	***P*** value
6 h	MCP-1	0.365	0.000	0.002
	eGFR baseline	−0.486	0.001	0.000
12 h	eGFR baseline	−0.493	0.001	0.000
24 h	MCP-1	0.301	0.000	0.014
	eGFR baseline	−0.353	0.001	0.004
3 days	IL-6	0.336	0.005	0.005
	MCP-1	0.282	0.000	0.016
	eGFR baseline	−0.359	0.001	0.003
7 days	IL-6	0.505	0.004	0.000
	MCP-1	0.236	0.000	0.023
	eGFR baseline	−0.316	0.001	0.003

Multiple stepwise linear regression analysis adjusted for age, gender, eGFR at baseline and stroke severity at admission. eGFR, estimated glomerular filtration rate; IL-6, interleukin-6; MCP-1, monocyte chemotactic protein-1; SDMA, symmetric dimethylarginine.

## Discussion

The aim of this study was to investigate the relationship between ADMA and SDMA as mediators of oxidative stress and endothelial dysfunction on the one hand and the inflammatory response on the other hand in patients with acute ischemic stroke.

After the acute event of stroke, the proinflammatory cytokine IL-6 is rapidly released by activated cells in brain tissue and peripheral blood [18]. In contrast to this, SDMA and ADMA levels increase during the first day. SDMA and ADMA are not correlated with IL-6 during the first 24 hours after stroke onset but we observed a correlation days after the event. Also in patients with coronary artery disease and chronic kidney disease, SDMA and IL-6 levels were correlated [19, 20]. Recently Tripepi *et al*. showed that increased levels of ADMA in combination with high IL-6 and CRP levels are predictive for death and cardiovascular events in patients with end-stage renal disease [21]. Cell culture experiments suggest a direct link between dimethylarginines and IL-6. SDMA has been shown to increase expression of IL-6 in monocytes, whereas in adipocytes ADMA triggers the expression of IL-6 via activation of nuclear factor-kappa B [22]. However, since levels of dimethylarginines peak when IL-6 has already significantly decreased during the first day after stroke, ADMA and SDMA are unlikely to induce the hyperacute increase of IL-6 in the first hours after stroke. Of note, in healthy young adults an inverse correlation of IL-6 with DDAH2 expression has been shown [23]. Decreased DDAH2 expression leads to an accumulation of ADMA in the human body. Thus in case of stroke, the relationship between IL-6 and ADMA is contrary to the observations in cell cultures. ADMA might increase at least in part as a response to high IL-6 levels.

In the current study, SDMA plasma levels were correlated with MCP-1 at the early stage but also days after the acute event independently of renal function. The chemokine MCP-1 recruits monocytes/macrophages to the injury site after stroke onset, which triggers the inflammatory reaction by further release of mediators [24]. Moreover, *in vitro* data suggest that SDMA triggers inflammation by enhancing reactive oxygen species (ROS) production of monocytes via modulation of store-operated calcium channels [25]. In contrast to SDMA, ADMA was not correlated to MCP-1 in this study, although ADMA has been shown to increase MCP-1 *in vitro*[26].

Considering data from the literature, both ADMA and SDMA levels are expected to increase in the state of chronic inflammation [27, 28] and to decrease in the state of acute inflammation. Zoccali *et al*. showed in 17 patients with bacterial infection that during the acute phase, patients displayed very high levels of CRP, IL-6, procalcitonin and nitrotyrosine [29]. When infection resolved, ADMA levels rose significantly while SDMA levels remained unmodified. After kidney donation of healthy subjects, ADMA levels temporarily decreased in the state of acute elevated inflammation [30]. Decline of the inflammation markers (IL-6 and CRP) was accompanied with an increase in ADMA levels. Further, Blackwell *et al*. showed that ADMA decreased rapidly during the first 48 hours of acute inflammation after total knee arthroplasty [31].

One explanation for the difference between stroke and other types of acute systemic inflammation might be the fact that both oxidative stress and inflammation are induced by the detrimental cascade after acute ischemic stroke. This may not prove the mechanistical association but at least that levels are increased in parallel. The missing correlation between dimethylarginines and the marker of the extent of brain cell damage S100B in the current study suggests that the increase of dimethylarginines is not primarily caused by the amount of degradation of malperfused brain tissue. Moreover, experimental data suggest that a link between ADMA levels and infarct size is lacking. Leypoldt *et al*. investigated the infarct size in a transient middle cerebral artery occlusion (tMCAO) model of human DDAH-1 (hDDAH-1) transgenic (TG) mice [32]. Size of infarction did not differ between hDDAH-1 TG mice and wild-type (WT) littermates. But also DDAH activity in the brain and cerebral ADMA tissue levels in TG mice remained unchanged. However, there may be differences between animal models and patients, since in a rat model of tMCAO ADMA levels in serum and brain were unchanged, while they were significantly decreased in cerebrospinal fluid (CSF) [33].

ADMA might be involved in post-ischemic recovery, as ADMA and TIMP-1 were independently correlated during the first hours and also days after the event. TIMP-1 is an endogenous MMP-9 inhibitor. Glial cell expression of TIMP-1 has been shown to be regulated by microglia [34]. TIMP-1 concentrations are elevated early after ischemic stroke onset [35]. In monocytes of 25 ischemic stroke patients, TIMP-1 expression was increased during the first days after the event [36]. Elevated levels of TIMP-1 might limit matrix proteolysis and thereby play a role in tissue remodelling [37]. Moreover, a possible involvement of ADMA in post-ischemic recovery is supported by the fact that ADMA was inversely correlated with MMP-9 at 12 hours after stroke onset in our study. MMP-9 is known as a member of the zinc-binding proteolytic enzymes, which degrades extracellular matrix (ECM) proteins found in the basal lamina of blood vessels. Thereby, increased MMP-9 levels are associated with haemorrhagic transformation after ischemic stroke [38]. In a mouse model of ischemic stroke, the nonselective NOS inhibitor N-omega-nitro-L-arginine (L-NA) decreased MMP-9 expression [39]. In a recently published study in rats, lowering of NO by the NOS inhibitor L-NG-nitroarginine methyl ester (L-NAME) levels led to reduced blood–brain barrier disturbance [40]. As a nonselective NOS inhibitor, ADMA might have similar effects, though we do not have direct evidence yet. Remarkably, MMP-9 levels were not elevated in stroke patients compared with those in controls in our study. At day 3, levels of MMP-9 in patients were even significantly lower. While several studies showed increased MMP-9 levels in stroke patients compared to controls [41–43], few studies did not detect clear differences [44, 45]. In accordance to our results, in two studies median levels of MMP-9 were - although not significantly - lower in stroke patients than in controls at 3 days and 2 to 5 days after stroke, respectively [45, 46]. Further studies need to clarify these controversial findings.

In addition to the shown association of dimethylarginines and inflammation, we cannot exclude an effect of pharmacological treatment on dimethylarginine levels in our patients. Some drugs lower dimethylarginine levels, such as statins, angiotensin-converting enzyme inhibitors and angiotensin receptor blockers [47, 48]. Metabolic changes like impaired glucose tolerance and stroke-associated neurohumoral changes might also have an effect on dimethylarginine levels. ADMA has repeatedly been shown to increase with high glucose levels [27], insulin resistance [49] and hyperthyroidism [50]. Although our analysis did not show an association between pharmacological treatment, metabolic changes and dimethylarginine levels, we cannot rule out an effect of these factors on the plasma levels of ADMA and SDMA since the patient cohort was too small for such an analysis. Furthermore, our data did not show differences in levels of dimethylarginines in regard to stroke etiology. This is in accordance with data reported by Brouns *et al*. who investigated ADMA levels in the CSF of patients with stroke [4]. In contrast, Scherbakov *et al*. found plasma concentrations of ADMA elevated predominantly in patients with atherothrombotic and cardioembolic stroke and not in those with lacunar infarction or undetermined cause of stroke [51]. In Swedish stroke patients, serum concentrations of ADMA were increased in cardioembolic stroke, but not in noncardioembolic stroke [3]. So far these differences have not been explained pathophysiologically. However, these controversial data may result from different patient populations.

Of note, in the present study SDMA levels were significantly higher in controls than in stroke patients at 6 hours. The concentration of SDMA largely depends on renal function as it is mostly excreted by the kidney. Therefore SDMA is a sensitive marker for slight renal impairment. Higher SDMA levels in controls suggested lower eGFR in this group. Comparison of the respective data, however, showed no significant difference. As expected SDMA concentrations in the patients were inversely correlated with eGFR at baseline at each time point. Similar results have been observed in other studies [29, 52]. It shall be emphasised, however, that according to the results of the multivariate regression analysis which included eGFR into the statistical model, the observed association between concentrations of SDMA and inflammatory markers in this study is independent of renal function.

Our data indicate for the first time an association between dimethylarginines as mediators of oxidative stress and inflammation after ischemic stroke. This link points to a potential role of dimethylarginines in the detrimental cascade in stroke pathophysiology and might partly explain the correlation of dimethylarginines with stroke outcome [6, 53].

However, our study has some limitations. It remains unclear whether elevated dimethylarginine levels are the cause or the result of increased inflammation, which deserves further investigation in experimental models. Furthermore, the number of patients included into this study was small. However, since we collected serial blood samples in short intervals after ischemic stroke we were able to describe the temporal change of levels of dimethylarginines and mediators of inflammation during the first days after the event, which has never been done before. Finally, only few severe strokes were included in this study. Since inflammation and oxidative stress strongly increase in severe strokes, inclusion of a large number of mild to moderate strokes in this study might have led to underestimation of the association between dimethylarginines and inflammatory mediators.

## Conclusions

Our data show that levels of ADMA were increased after acute ischemic stroke, while levels of its structural isomer SDMA were even decreased in the first hours. Both ADMA and SDMA, known as markers of cardiovascular risk and outcome, are associated with the inflammatory response in acute ischemic stroke patients. However, to explain the link between dimethylarginines and inflammation in stroke pathophysiology further investigations are needed.
