# Correction: Association of dimethylarginines and mediators of inflammation after acute ischemic stroke

**DOI:** 10.1186/s12974-023-02775-0

**Published:** 2023-05-03

**Authors:** Shufen Chen, Jens Martens-Lobenhoffer, Karin Weissenborn, Jan T. Kielstein, Ralf Lichtinghagen, Milani Deb-Chatterji, Na Li, Anita B. Tryc, Annemarie Goldbecker, Qiang Dong, Stefanie M. Bode-Böger, Hans Worthmann

**Affiliations:** 1 Department of Neurology, Hannover Medical School, Carl-Neuberg-Strasse 1, 30623 Hannover, Germany; 2 Department of Neurology, Huashan Hospital, Fudan University, 12 Wulumuqi Zhong Road, Shanghai, 200040 China; 3 Department of Clinical Pharmacology, Otto-Von-Guericke University of Magdeburg, University Hospital, Leipziger Strasse 44, 39120 Magdeburg, Germany; 4 Center for Systems Neuroscience (ZSN), Bünteweg 2, 30559 Hannover, Germany; 5 Department of Nephrology and Hypertension, Hannover Medical School, Carl-Neuberg-Strasse 1, 30623 Hannover, Germany; 6 Department of Clinical Chemistry, Hannover Medical School, Carl-Neuberg-Strasse 1, 30623 Hannover, Germany; 7 Department of Neurology, Beijing Tiantan Hospital, Capital Medical University, 6, Tiantan Xili, Beijing, 100050 China

**Correction: Journal of Neuroinflammation 2012, 9:251** **http://www.jneuroinflammation.com/content/9/1/251**

Following publication of the original article [1], the co-author Dr. Milani Deb-Chatterji would like to update the family name from "Deb" to "Deb-Chatterji.

Hence, her name has been updated in this correction article.
